# 

**Published:** 1866-02

**Authors:** 


### Books and Pamphlets Received.

Transactions of the Medical Society of the State of New York, for the year 1865.

The Physiology of Man; designed to represent the existing state of Physiological Science, as applied to the functions of the human body, by Austin Flint, jr., M. D. New York: D. Appleton & Co., 1866.

A Treatise on the Principles and Practice of Medicine; designed for the use of practitioners and students of medicine, by Austin Flint, M. D. Philadelphia: Henry C. Lea, 1866.

An Appeal by the Citizens' Asssociation of New York, against the abuses in the local government, to the Legislature of the State of New York, and to the public.

Communication from the Governor transmitting the Report of the Commissioners of Quarantine. Transmitted to the Legislature February 16, 1865.

Twelfth Annual Report of the Trustees of the State Lunatic Hospital at Taunton, Mass.

An account of the proceedings at the laying of the Corner Stone of the State Emigrant Hospital, on Ward's Island, August 10, 1864. Address with statistics by Gulian C. Verplanck.

Drake's Reporter of Artificial Limbs, etc., etc., 1859–60.

Contributions to Bone and Nerve Surgery, by J. C. Nott, M. D. Philadelphia: J. B. Lippincott & Co., 1866.

New Medical Journal in Cincinnati, Ohio.—We have the pleasure of adding to our exchange list *The Cincinnati Journal of Medicine*, January number, Vol. I, No. 1, being upon our table. It is published monthly, contains forty-eight pages, and has the following able editorial staff: George C. Blackman, M. D., Theophilus Parvin, M. D., Roberts Bartholow, M. D., Professors in the Medical College of Ohio.

### American Medical Association.

The Seventeenth Annual Session will be held in the city of Baltimore, on Tuesday, May 1, 1866.

The following Committees are expected to report:

On Prize Essays, Dr. Austin Flint, Sr., New York, Chairman.

On Quarantine, Dr. Wilson Jewell, Pa., Chairman.

On So-called Spotted Fever, Dr. Jas. J. Levick, Pa., Chairman.

On Ligature of the Subclavian Artery, Dr. William Parker, N. Y. Chairman.

On Tracheotomy in Membranous Croup, Dr. Alex. N. Dougherty, N. J., Chairman.

On Rank of Medical Corps in the Army, Dr. C. S. Tripler, U. S. A., Chairman.

On Rank of Medical Corps in the Navy, Dr. T. L. Smith, N. Y. Chairman.

On Medical Literature, Dr. C. A. Lee, N. Y., Chairman.

On Medical Education, Dr. Samuel D. Gross, Pa., Chairman.

On American Necrology, Dr. C. C. Cox, Md., Chairman,

On Patent Rights and Medical Men, Dr. David Prince, Ill., Chairman.

On Alcohol and its Relations to Man, Dr. Gerard E. Morgan, Md., Chairman.

On Insanity, Dr. Alfred Hitchcock, Mass., Chairman.

On Milk Sickness, Dr. Robert Thompson, Ohio, Chairman.

On the relation which the Doctrine of the Correlation and Conservation of Forces bears to the Physiological and Pathological Condition of the Human System, Dr. S. L. Loomis, D. C., Chairman.

On the Progress of Medical Science, Dr. Jerome Candee Smith, N. Y., Chairman.

On Diphtheria, Dr. H. D. Holton, Vt., Chairman.

On the Comparative Value of Life in City and Country, Dr. Edw. Jarvis, Mass., Chairman.

On Drainage and Sewerage of Cities in their Influence on Health, Dr. Wilson Jewell, Pa., Chairman.

What Effects has Civilization on the Duration of Human Life, Dr. Augustus A. Gould, Mass., Chairman.

On Disinfectants. Dr. E. M. Hunt, N. J., Chairman.

On Compulsory Vaccination, Dr. A. Nelson Bell, N. Y. Chairman.

On Strangulated Hernia, Dr. W. F. Peck, Iowa, Chairman.

On the Causes and Pathology of Pyæmia, Dr. J. J. Woodward, U.S.A., Chairman.

On the Use of Plaster of Paris in Surgery, Dr. Jas. L. Little, N. Y., Chairman.

On the Etiological and Pathological Relations of Epidemic Erysipelas, Spotted Fever, Diphtheria, and Scarlatina, Dr. N. S. Davis, Ill., Chairman.

On Meteorology, Medical Topography, and Epidemics:

| | |
|---|---|
| Dr. J. C. Weston, Me. | Dr. D. Francis Condie, Pa. |
| " P. A. Stackpole, N. H. | " T. Antisell, D. C. |
| " C. L. Allen, Vt. | " O. S. Mahon, Md. |
| " A. C. Garratt, Mass. | " T. M. Logan, Cal. |
| " C. W. Parsons, R. I. | " R. C. Hamill, Ill. |
| " B. H. Catlin, Conn. | " J. W. H. Baker, Iowa. |
| " E. M. Chapman, N. Y. | " Abm. Sager, Mich. |
| " E. M. Hunt, N. J. | " J. W. Russell, Ohio. |

WILLIAM B. ATKINSON,
Permanent Secretary, Philadelphia.

